# Bioceramics Based on Li-Modified Bioactive Glasses for Bone Tissue Regeneration

**DOI:** 10.3390/ma19010153

**Published:** 2026-01-01

**Authors:** Mihai Fotu, Adrian Ionuț Nicoară, Ștefan Manolache, Mihaela Bacalum, Roberta Moisa (Stoica), Roxana Doina Trușcă, Gabriela Olimpia Isopencu, Cristina Busuioc

**Affiliations:** 1 Department of Science and Engineering of Oxide Materials and Nanomaterials, Faculty of Chemical Engineering and Biotechnologies, National University of Science and Technology Politehnica Bucharest, 1-7 Gh. Polizu, 060042 Bucharest, Romania; mihai.fotu@stud.chimie.upb.ro (M.F.); cristina.busuioc@upb.ro (C.B.); 2 National Research Center for Micro and Nanomaterials, National University of Science and Technology Politehnica Bucharest, 313 Spl. Independenţei, 060042 Bucharest, Romania; truscaroxana@yahoo.com; 3 Faculty of Medical Engineering, National University of Science and Technology Politehnica Bucharest, 1-7 Gh. Polizu, 060042 Bucharest, Romania; manolache.stefan99@gmail.com; 4 Department of Life and Environmental Physics, Horia Hulubei National Institute of Physics and Nuclear Engineering, Strada Reactorului 30, 077125 Măgurele, Romania; bmihaela@nipbe.ro (M.B.); roberta.stoica@nipne.ro (R.M.); 5 Department of Chemical and Biochemical Engineering, Faculty of Chemical Engineering and Biotechnologies, National University of Science and Technology Politehnica Bucharest, 1-7 Gh. Polizu, 060042 Bucharest, Romania; gabriela.isopencu@upb.ro

**Keywords:** lithium-modified bioglass, bone tissue engineering, antibacterial activity, structural characterization, mechanical properties, simulated body fluid, combeite, lithium silicate

## Abstract

The development of effective bone substitutes remains a central goal in regenerative medicine. In this study, lithium-modified bioglass-ceramics based on the 47.5S5 silicate oxide system were synthesized using the sol–gel method, followed by calcination and axial pressing to form cylindrical samples. These materials were sintered at 700 and 800 °C and subsequently examined to evaluate their structural, mechanical, and biological performance. Structural and microstructural analyses confirmed the presence of crystalline phases such as combeite (Na_6_Ca_3_Si_6_O_18_), NaLiSiO_4_, Li_2_SiO_3_, and calcium silicates, indicating the successful incorporation of lithium within the glass-ceramic network. The bioceramics exhibited improved densification, deformability, and compressive strength with increasing sintering temperature. In vitro degradation in simulated body fluid revealed a consistent increase in mass loss with higher lithium content, suggesting enhanced resorbability linked to lithium oxide. Antibacterial testing indicated moderate antimicrobial activity, with slightly better results observed at higher sintering temperatures. Cell viability assays further supported the materials cytocompatibility. Taken together, these findings suggest that lithium substitution contributes positively to both mechanical robustness and biological behaviour, positioning these ceramics as promising bioresorbable bone substitutes with controlled degradation, suitable for bone tissue engineering where durability, bioactivity, and antimicrobial function are required.

## 1. Introduction

Diseases affecting bone tissue and trauma-induced defects, such as osteoporosis, osteosarcoma, and critical-size fractures [1,2,3] remain major global health problems with profound clinical and socioeconomic impacts. Despite bone intrinsic regenerative capacity, severe injuries often exceed its ability to self-repair, requiring complex medical interventions. Traditional treatments, including autografts and donor bone grafts, face significant limitations such as limited availability [2,4], immune rejection, and side effects at the place where the grafts were collected, which drive the need for alternative therapeutic strategies. In this context, regenerative medicine has increasingly turned toward biomaterials [5,6,7] to support and enhance bone healing. Among these, bioactive bioglass-ceramics have gained considerable attention due to their ability to be customized in composition, their good biocompatibility [1,2,5,8], and their capacity to bond directly with bone by forming an apatite layer [9] under physiological conditions. These glass–ceramics are often derived from or closely related to bioactive glasses, which can be formulated as silicate-, borate-, and phosphate-based systems [3]. The specific glass composition strongly influences how the material behaves in the body, determining its interaction with tissues and its ability to support healing. These materials can be further improved by adjusting their composition or adding small amounts of certain ions [6,8] to boost their performance, such as bone bonding ability, strength, antibacterial activity [10,11] and cell viability [2,12]. Bioglass-ceramic materials are widely used in bone tissue regeneration due to their mechanical properties that closely resemble those of natural bone, including compressive strength, low elasticity, and structural stability [13]. Their excellent performance is also attributed to their chemical and structural similarity to the mineral phase of bone [14,15].

Since the introduction of 45S5 Bioglass^®^ [16,17,18,19], a silicate-based bioactive glass with a nominal oxide composition of SiO_2_–CaO–Na_2_O–P_2_O_5_, numerous compositions have been developed by adjusting the ratio or type of oxides with distinct dissolution rates and biological responses. These bioglasses can be synthesized via traditional melt-quenching or sol–gel methods [16,18,20], the latter allowing better control over porosity and surface reactivity. Among the various silicate-based bioactive glass formulations reported in the literature, the 47.5S5 composition (47.5 wt% SiO_2_, 23.75 wt% CaO, 23.75 wt% Na_2_O, and 5 wt% P_2_O_5_) has attracted particular attention due to its balanced bioactivity, controlled degradation behaviour, and suitability for bone tissue regeneration applications [21,22]. In addition to classical formulations, lithium-substituted bioactive glasses [20,23,24] have been investigated for their potential to enhance osteogenesis and influence glass structure through network modification [25,26].

Furthermore, controlled thermal treatments (650–950 °C) [27,28] can induce partial crystallization, leading to glass-ceramics with improved mechanical strength [24,27] and optimized properties. The nature and proportion of crystalline phases formed during sintering depend on both composition and temperature [16,28], enabling the design of materials suited for specific clinical needs in hard tissue repair.

Successful bone regeneration requires materials that combine structural support, biological stimulation, and protection against infection. Lithium-modified bioactive glasses have been proposed as multifunctional scaffolds [20,25,26,29,30] capable of responding to these complex needs. For instance, bone tissue repair depends on materials that can stimulate cell proliferation and support new bone formation [30,31,32]. Lithium ions have been reported to enhance osteogenesis [20,33] and angiogenesis, both essential for defect healing and vascular integration [30,31]. For example, Fatima Gomez Gramajo et al. [24] showed that glass-ceramic microparticles based on 45S5 bioglass, partially modified with 5 wt% Li_2_O (45S5.5Li), exhibited antioxidant activity and stimulated bone formation in vivo under both physiological and experimental conditions, highlighting their potential as inorganic osteogenic agents in bone regenerative medicine. From a structural standpoint, bone scaffolds must also withstand physiological loads. Due to its small ionic radius, lithium contributes to the compaction of the silicate network in bioglass, improving densification [30,32], microhardness, and mechanical integrity [32]. Additionally, open fractures and surgical procedures carry a high risk of infection [34], which may compromise healing and lead to chronic complications. Lithium-modified materials have demonstrated enhanced antibacterial activity [35,36] against oral and bone-related pathogens such as S. aureus, S. mutans, and P. gingivalis, by increasing pH and inhibiting bacterial adhesion [35,36,37,38]. Lastly, for any scaffold to be clinically relevant, it must be biocompatible. Lithium-containing glasses and ceramics have shown favourable cellular responses in vitro, supporting osteoblast viability and proliferation [29,37,38]. Altogether, these findings support the integration of lithium ions into bioactive glass formulations as a strategy to address the multiple challenges of bone tissue engineering.

In this study, a novel approach was employed by developing vitroceramic bulk samples based on lithium-modified bioglass powders, a substitution rarely explored in this context, and sintered at two temperatures of 700 and 800 °C. These conditions were selected with the goal of preserving the material bioactive potential while achieving sufficient mechanical integrity and maintaining an open porous structure suitable for bone tissue ingrowth. The resulting materials were extensively analysed to assess their structural, morphological, and biological characteristics. This evaluation aimed to determine their suitability as bioactive scaffolds for use in bone tissue engineering, where both porosity and mechanical strength are critical factors for clinical success.

## 2. Materials and Methods

### 2.1. Processing of Bioglass-Based Materials

The lithium-modified bioglass materials were designed to obtain a target oxide composition based on the 47.5% SiO_2_, 23.75% CaO, 23.75% Na_2_O, and 5% P_2_O_5_, with partial replacement of CaO by 2.5, 5, and 10 wt% Li_2_O (the full compositional details of each formulation are presented in Table 1) The sol–gel method was employed, using calcium nitrate (Ca(NO_3_)_2_∙4H_2_O, *p* ≥ 98%, Sigma-Aldrich, St. Louis, MO, USA), sodium nitrate (NaNO_3_, *p* ≥ 98%, Sigma-Aldrich, St. Louis, MO, USA), lithium sulphate (Li_2_SO_4_, *p* ≥ 98%, Sigma-Aldrich, St. Louis, MO, USA), tetraethyl orthosilicate (TEOS-(C_2_H_5_O)_4_Si, *p* ≥ 99%, Sigma-Aldrich, St. Louis, MO, USA), and triethyl phosphate (TEP-(C_2_H_5_O)_3_PO, *p* ≥ 99.8%, Sigma-Aldrich, St. Louis, MO, USA) as precursors.

Synthesis of the initial powders followed the route described by Fotu et al. [20] and began with the dissolution of the solid precursors in distilled water and ethanol, followed by homogenization under magnetic stirring at 60 °C for approximately 30 min. Under continuous stirring, the liquid precursors were added dropwise, and the solution was further stirred for about 1 h at 60 °C until gel formation occurred. The resulting gel was then dried in an oven at 80 °C for 24 h to ensure complete solvent evaporation. After drying, the material was finely ground and subsequently calcined at 600 °C for 1 h, leading to the formation of a lithium-modified bioglass powder suitable for further processing [20]. The starting bioglass powders showed a fine particle size distribution and a glass-ceramic structure dominated by combeite, with additional lithium silicate phases, as detailed in our previous work. In the present work, we focus on the subsequent processing and characterization of the sintered bioceramic materials derived from these powders.

**Figure 1:**
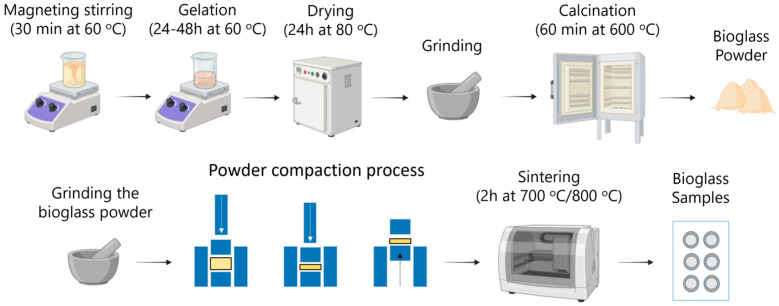
Schematic representation of the fabrication process for lithium-modified biomaterials: sol–gel synthesis, calcination at 600 °C, powder compaction, and sintering at 700 °C/800 °C.

This powder was manually ground to a fine consistency and compacted by uniaxial pressing into cylindrical green bodies. An initial evaluation of the thermal behaviour was carried out by sintering test samples at 700, 800, 900, and 1000 °C. The samples sintered at temperatures above 800 °C exhibited partial melting and loss of shape, indicating thermal instability at higher temperatures. As a result, the working temperatures were limited to 700 and 800 °C, which allowed adequate consolidation without compromising the geometry or surface integrity of the pellets. The overall experimental approach followed for obtaining the sintered materials is illustrated schematically in Figure 1.

The final samples were labelled according to their lithium content and processing temperature. These materials were subsequently subjected to detailed structural, morphological, and biological evaluations to assess their potential as bioactive scaffolds in bone tissue regeneration.

### 2.2. Characterisation Techniques and Instrumentation

The structural properties were obtained at room temperature using X-ray diffraction (XRD) equipment with a Cu X-ray tube (*λ* CuKα1 = 1.541874 Å) provided by PANalytical Empyrean system (Melvern-PANalytical, Almelo, The Netherlands). Scans were performed between 5 and 80° with 0.02° increments and a dwell time of 100 s per step.

Chemical bond identification and local structural arrangement of the samples were carried out at room temperature using Raman spectroscopy. The measurements were performed using a LabRAM HR Evolution spectrometer from Horiba (Palaiseau, France). Raman spectra were obtained for 2 s acquisition time and 20 accumulations, using a 514 nm argon-ion laser with a beam power of 125 mW.

The samples morphology was examined through scanning electron microscopy (SEM) using a Quanta Inspect F50 microscope paired with an energy dispersive spectrometer (EDS) (Thermo Fisher, Eindhoven, The Netherlands).

The ceramic properties (apparent density and open porosity) were evaluated using the Archimede’s method and calculated using Equations (1) and (2). The samples are weighed in air and placed in a vacuum desiccator. After vacuuming for 15 min (time required to remove the air from the pores), the samples are immersed in a liquid (toluene). Subsequently, the samples saturated in toluene are extracted and weighed again, both in air and then in toluene.

(1) $$apparent\;density=\frac{w_{i}\cdot \rho_{t}}{w_{ta}-w_{t}}$$

(2) $$open\;porozity=\frac{w_{ta}-w_{i}}{w_{ta}-w_{t}}$$

where *w_i_*—the initial weight of the sample (g); *w_ta_*—the weight (measured in air) of the liquid impregnated sample (g); *w_t_*—the weight (measured in toluene) of the liquid impregnated sample (g); *ρ_t_*—toluene density (0.867 g/cm^3^).

The thermal shrinkage was calculated by measuring the cylindrical samples using a caliper. The measurements were performed before and after the sintering process so that the shrinkage assessment could be performed as a function of volume. Height (h) and diameter (d) were measured and then Equations (3) and (4) were applied. The weight loss was similarly measured, considering the weight of the sample before and after sintering.

(3) $$weight\;loss=\frac{w_{i}-w_{f}}{w_{i}}\cdot 100$$

(4) $$shrinkage=\frac{V_{i}-V_{f}}{V_{i}}\cdot 100$$

where *w_i_*—the weight of the sample before sintering (g); *w_f_*—the weight of the sample after sintering (g); *V_i_*—the volume of the sample before sintering (cm^3^); *V_f_*—the volume of the sample after sintering (cm^3^).

To assess stability in a wet environment, pieces from each composition studied were placed in approximately 5 mL of simulated body fluid (SBF) with a pH of 7.4, prepared according to the Kokubo protocol [39]. The initial mass of each sample was recorded, after which the samples were immersed in the SBF solution. At predetermined intervals of 7, 14, and 28 days, the samples were removed, dried, and weighed again. The mass loss could be calculated using Equation (5):

(5) $$mass\;loss=\frac{w_{i}-w_{f}}{w_{i}}\cdot 100$$

where *w_i_* = sample weight before immersion and *w_t_* = dried sample weight after *t* min of immersion in SBF.

The mechanical compressive strength was evaluated by compressing the samples to the breaking point at a speed of 1 mm/min using the Shimadzu Autograph AGS-X 20kN equipment (Shimadzu, Tokyo, Japan).

All tests were performed in triplicate for each ceramic obtained and data are presented as means values ± standard error from the triplicate analysis.

The antibacterial properties of the synthesized samples were evaluated using a diffusion-based inhibition zone assay. Gram-negative bacteria (*Escherichia coli*, DH5K strain) and yeast (*Candida albicans*) were used as model microorganisms, grown on LB (Miller, Sigma-Aldrich, St. Louis, MO, USA) agar and Sabouraud Dextrose Agar (SDA), respectively. The agar plates were inoculated with 1 mL of microbial suspension adjusted to an optical density (OD_600_) of 0.558 for *E. coli* and 0.456 for *C. albicans*, using the flooding method. Excess inoculum was removed, and the plates were pre-incubated for 1 h at controlled humidity to ensure uniform impregnation. The samples were sterilized under UV light (256 nm, ROTH UV lamp type IV 254/366 nm) for 30 min, and then aseptically placed on the agar surface. Plates were incubated for 24 h at 37 °C, and the antimicrobial activity was quantified by measuring the diameter of the inhibition zone (IZ, expressed in mm).

The biocompatibility of the synthesized materials was assessed using the MTT assay on MG-63 human osteoblast-like cells (ATCC^®^ CRL1427). Cells were cultured in DMEM medium supplemented with 10% FBS and 1% penicillin/streptomycin and incubated at 37 °C with 5% CO_2_. Samples were dissolved in sterile medium to a final concentration of 25 mg/mL. After seeding in 96-well plates (7000 cells/well), cells were treated with different sample concentrations for 24 and 48 h. Viability was determined by replacing the medium with MTT solution (1 mg/mL), followed by 4 h incubation. Formazan crystals were solubilized in DMSO, and absorbance was measured at 590 nm using a Mithras LB 970 microplate reader (Berthold Technologies, Bad Wildbad, Germany). Viability (%) was calculated as the ratio between sample and control absorbance values.

Cell morphology was evaluated by fluorescence microscopy. Cells were fixed with 3% paraformaldehyde, permeabilized with Triton X-100, and stained with FITC-phalloidin to visualize actin filaments. Nuclei were counterstained with Hoechst 33342. Slides were mounted using FluorSave^TM^ (Sigma-Aldrich, St. Louis, MO, USA) and imaged using an Olympus BX-51 epifluorescence microscope (Olympus, Hamburg, Germany) equipped with a 40× objective and DAPI/Hoechst and GFP/FITC filters.

## 3. Results and Discussion

### 3.1. Morpho-Structural Analysis

**Figure 2:**
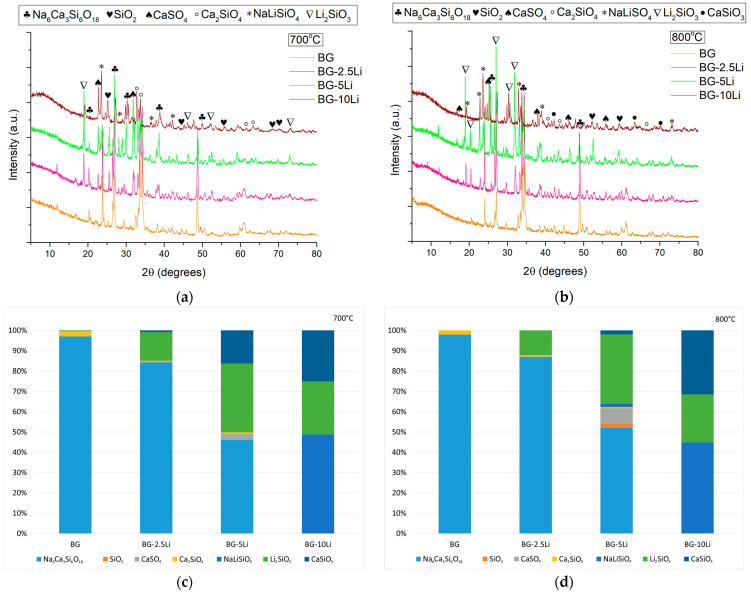
XRD patterns of lithium-modified bioglass samples sintered at 700 °C (**a**) and 800 °C (**b**), corresponding to BG, BG-2.5Li, BG-5Li, and BG-10Li and quantitative phase composition determined by Rietveld refinement of the XRD data for the samples at 700 °C (**c**) and 800 °C (**d**).

The XRD patterns of the sintered ceramics shown in Figure 2a,b indicate the formation of multiple crystalline phases.

All samples exhibit reflections corresponding to sodium calcium silicate (Na_6_Ca_3_Si_6_O_18_—PDF5 04-012-8759) known as combeite, silica (SiO_2_—PDF5 04-025-3507), calcium sulphate (CaSO_4_—PDF5 00-003-0163), calcium silicate (Ca_2_SiO_4_—PDF5 04-006-2363), and sodium lithium silicate (NaLiSiO_4_—PDF5 04-016-0897), compounds that are frequently reported in the literature across various glass-ceramic systems due to their beneficial structural and biological roles, thereby supporting the classification of these materials as bioactive glass-ceramics suitable for biomedical applications [40,41,42,43,44]. A noticeable increase in the intensity of peaks attributed to combeite and lithium metasilicate (Li_2_SiO_3_—PDF5 00-029-0828) is observed with higher sintering temperature, especially in the 800 °C samples, indicating the development of a glass-ceramic structure with a higher proportion of crystalline phases. In addition, a broad halo can be observed in the low-angle region of the diffractograms, which indicates the presence of a residual amorphous component. This feature is consistent with the glassy nature of the sol–gel derived powders and suggests that part of the amorphous matrix is still retained after sintering. Because of the inherent difficulty of quantifying this amorphous contribution, the Rietveld refinement was performed only on the crystalline phases.

Within this framework, the quantitative Rietveld refinement confirms this trend, showing a gradual decrease in Na_6_Ca_3_Si_6_O_18_ content from ~95% in BG to less than 50% in BG-10Li, with a corresponding increase in Li_2_SiO_3_ and CaSiO_3_ phases, especially at 800 °C. As illustrated in Figure 2c,d, BG-5Li and BG-10Li exhibit the most complex phase composition, with up to six distinct crystalline phases.

An enhancement in the amount of calcium sulphate is also evident in the materials, particularly with increased lithium content. This phase is known to promote tissue regeneration and improve biological integration in biomedical contexts [41,45]. Furthermore, the presence of lithium silicate in the structure—especially in the BG-10Li samples—may contribute to improved properties in dental applications due to its mechanical and biological performance [46,47,48].

**Figure 3:**
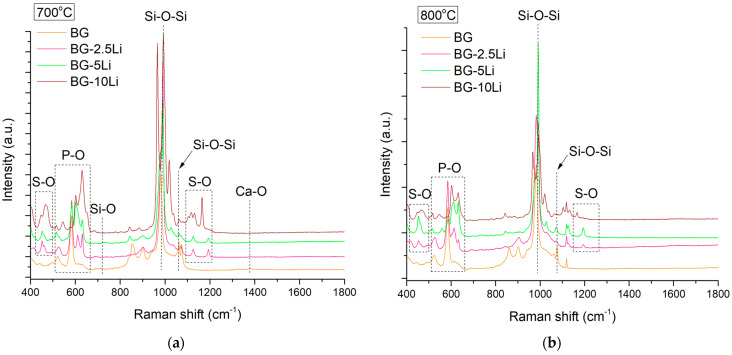
Raman spectral profiles of lithium-modified bioglass samples sintered at 700 °C (**a**) and 800 °C (**b**), including BG, BG-2.5Li, BG-5Li and BG-10Li.

The Raman spectra in Figure 3a,b show characteristic bands of silicate-based glass-ceramics.

The intense peak near ~1000 cm^−1^ corresponds to Si-O-Si symmetric stretching [49]. A subtle but noticeable increase in peak sharpness and intensity at 800 °C, especially for the 10-Li sample, suggests enhanced structural ordering and crystallinity with increasing lithium content and sintering temperature. An increase in peak intensity and sharpness with lithium content—especially at 800 °C—suggests enhanced structural organization and crystallinity [50].

Bands in the 400–700 cm^−1^ region are assigned to P-O and S-O vibrations, confirming the incorporation of phosphate and sulphate groups [51]. Overall, lithium substitution and higher sintering temperatures promote the development of a more ordered and bioactive glass-ceramic matrix, consistent with the XRD data.

**Figure 4:**
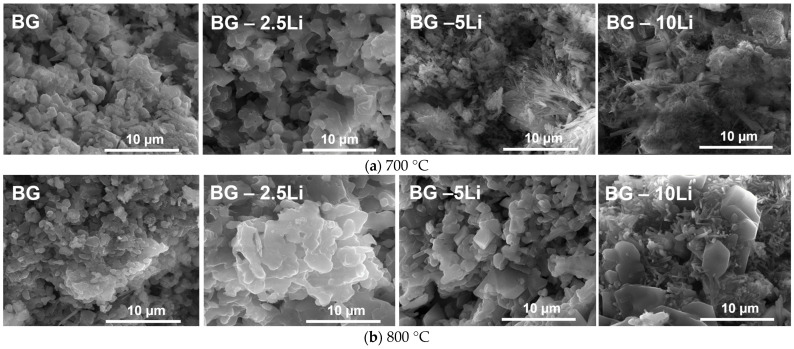
SEM images of lithium-modified bioglass samples sintered at 700 °C (**a**) and 800 °C (**b**), including BG, BG-2.5Li, BG-5Li and BG-10Li, recorded at a magnification of 10,000×.

The SEM micrographs in Figure 4a,b reveal significant morphological differences between the samples sintered at 700 and 800 °C, as well as across lithium content levels. At 700 °C, the BG and 2.5-Li samples display a rough and granular surface with poorly defined grains, indicating partial densification [24,52]. In contrast, the 5-Li and 10-Li samples show the formation of elongated crystalline structures and needle-like phases, suggesting lithium role in promoting local crystallization and phase separation at lower sintering temperatures.

At 800 °C, all samples exhibit improved densification, with more compact and better-defined crystal morphologies [53,54]. The 2.5-Li and 5-Li samples show stacked, plate-like crystallites, while the 10-Li sample reveals a mixture of dense regions and fibrous outgrowths, likely associated with enhanced crystal growth and phase development.

Overall, increasing lithium content and sintering temperature leads to more organized microstructures, supporting the formation of bioactive glass-ceramics with tailored surface features for biomedical applications [55].

**Figure 5:**
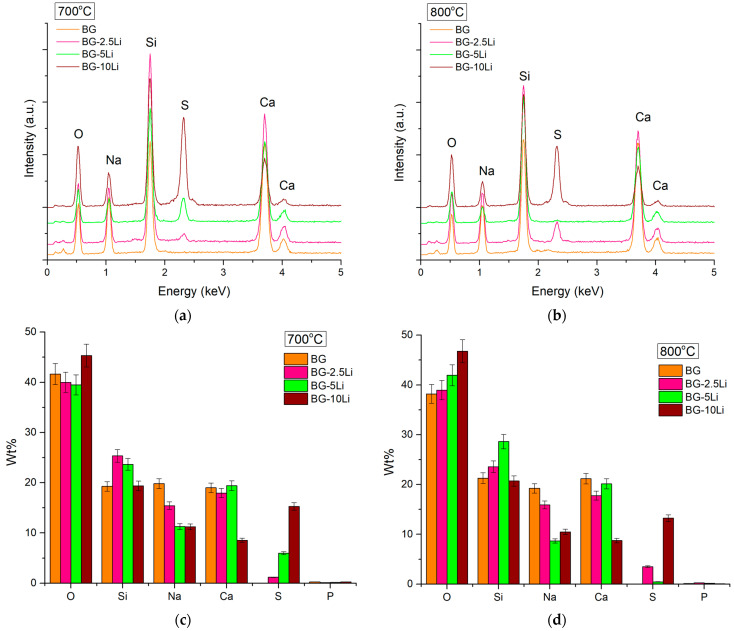
EDS spectra of lithium-modified bioglass samples sintered at 700 °C (**a**) and 800 °C (**b**) and Elemental composition (wt%) of the same samples at 700 °C (**c**) and 800 °C (**d**).

The EDS spectra, presented in Figure 5, confirm the presence of the main constituent elements of the glass-ceramic system, including oxygen (O), sodium (Na), silicon (Si), phosphorus (P), sulphur (S), and calcium (Ca).

The elemental peaks are consistent across all samples, with increasing intensity for Si and Ca in the 10Li sample, which may indicate a higher degree of crystallinity. Quantitative analysis (Figure 5c,d) shows a progressive increase in oxygen and sulphur content with higher lithium content, while silicon and sodium levels decrease, especially in the BG-10Li sample sintered at 800 °C. The detection of sulphur and phosphorus supports the incorporation of sulphate and phosphate groups, in agreement with Raman and XRD analyses, and may contribute to the bioactive potential of the material.

### 3.2. Ceramic and Mechanic Properties Evaluation

**Figure 6:**
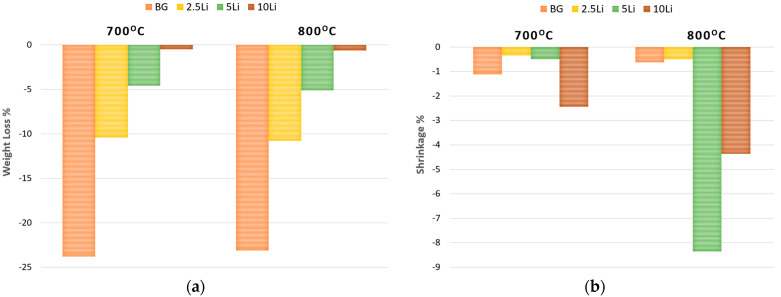
(**a**) Weight loss (%) and (**b**) volume shrinkage (%) of lithium-modified bioglass samples (BG, BG-2.5Li, BG-5Li and BG-10Li) sintered at 700 and 800 °C.

Figure 6a shows the percentage of material degradation after sintering, indicating a clear trend of decreasing weight loss with increasing lithium content.

While the base glass (BG) and 2.5-Li samples show the highest mass loss—up to ~24%—the 5-Li and especially 10-Li samples exhibit significantly lower values, suggesting that lithium incorporation enhances thermal stability. This behaviour may be attributed to the formation of more robust crystalline phases that reduce volatilization or decomposition during sintering. This effect aligns with previous findings by Xiong et al. [56], who demonstrated that higher lithium slag content promotes liquid-phase formation and densification in ceramic matrices, and is further supported by Zhao et al. [57], who reported improved sintering behaviour and reduced structural degradation in lithium-based ceramics processed via microwave sintering.

In Figure 6b, the shrinkage percentage reflects the densification behaviour during sintering. At 800 °C, the 5-Li sample exhibits the highest shrinkage (~8.5%), indicating significant structural compaction and potential crystal growth. The 10Li sample also shows notable shrinkage at 800 °C, suggesting that increased lithium promotes densification at higher temperatures. Overall, both degradation and shrinkage data support the role of lithium in modifying the thermal response and microstructural evolution of the glass-ceramic system.

**Figure 7:**
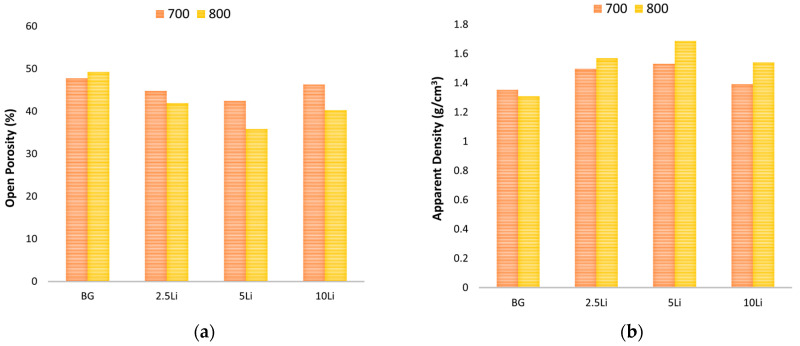
(**a**) Open porosity (%) and (**b**) apparent density (g/cm^3^) of lithium-modified bioglass samples (BG, BG-2.5Li, BG-5Li and BG-10Li) sintered at 700 and 800 °C.

The results in Figure 7a,b highlight the influence of lithium content and sintering temperature on the open porosity and apparent density of the sintered glass-ceramic samples.

A clear inverse relationship is observed between porosity and density: as lithium content increases, open porosity decreases, while apparent density increases, particularly in samples sintered at 800 °C. This trend suggests that lithium promotes densification, which leads to more efficient pore closure and particle rearrangement [58,59]. The 5Li sample sintered at 800 °C exhibits the highest apparent density and the lowest porosity among all groups, indicating optimal structural compaction. These findings are consistent with the XRD results, which showed an increase in crystalline phase content (such as Li_2_SiO_3_ and CaSiO_3_) and a decrease in amorphous or Na_6_Ca_3_Si_6_O_18_ content at higher lithium concentrations, supporting the conclusion that lithium enhances structural densification. These findings confirm the role of lithium in improving the microstructural integrity and packing efficiency of bioactive glass-ceramics.

**Figure 8:**
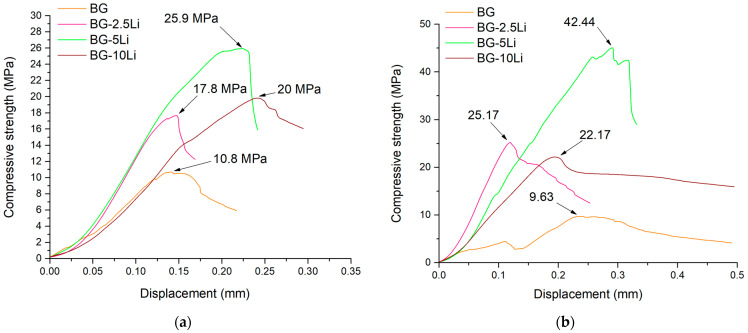
Compressive strength versus displacement curves of lithium-modified bioglass samples (BG, BG-2.5Li, BG-5Li and BG-10Li) sintered at 700 °C (**a**) and 800 °C (**b**).

The compressive strength results shown in Figure 8a,b demonstrate a significant enhancement in mechanical performance with lithium incorporation, particularly for the 5-Li composition.

At 700 °C, the compressive strength increases from 10.8 MPa for BG to 25.9 MPa for BG-5Li, indicating that moderate lithium substitution promotes structural integrity through improved densification and crystalline phase formation. The BG-10Li sample also shows a notable strength improvement, although slightly lower than 5Li, suggesting that excessive lithium content may reduce the mechanical benefits observed at intermediate concentrations [60].

At 800 °C, the trend is further amplified, with BG-5Li reaching the highest compressive strength of 42.44 MPa, almost double compared to the unsubstituted BG sample (9.63 MPa). This enhancement can be attributed to the synergistic effect of lithium and higher sintering temperature in promoting compact microstructures and mechanically resilient crystalline phases. These results confirm the beneficial role of lithium in optimizing the mechanical properties of sintered glass-ceramics.

As also reported by Li et al. [6] and Ye et al. [60], lithium disilicate-based glass-ceramics demonstrate excellent mechanical behaviour and bioactivity, largely attributed to their ability to undergo ion exchange and develop crystalline phases that improve structural integrity.

In this context, the presence of a small number of Li_2_SiO_3_ crystallites can induce radial compressive stress, which may help deflect or arrest crack propagation. Lithium, particularly in its disilicate form, plays a key role in enhancing the mechanical strength of the material through ionic exchange mechanisms, making it a promising candidate for dental and bone repair applications.

### 3.3. In Vitro Behaviour

**Figure 9:**
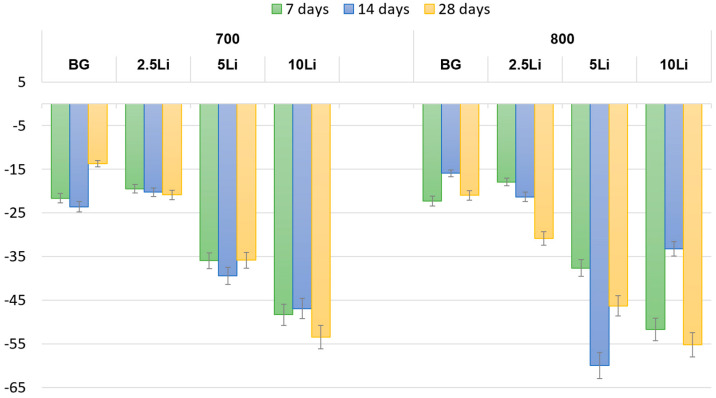
Degradation in SBF of bioglass (BG) and lithium-modified bioglass samples (2.5, 5 and 10 wt% Li) sintered at 700 and 800 °C, measured after 7, 14 and 28 days of immersion.

The degradation profiles shown in Figure 9 indicate a progressive mass loss over time for all samples, as expected for bioactive materials in SBF.

A general trend of increased degradation with immersion time is observed, particularly for samples sintered at 700 °C, which exhibit higher mass loss compared to those treated at 800 °C. This behaviour is associated with lower structural densification and increased surface reactivity at the lower sintering temperature. Notably, samples with higher lithium content (especially 5-Li and 10-Li) also show greater degradation, suggesting enhanced ion release dynamics.

At 800 °C, although sintering leads to improved structural consolidation, samples with high Li content still display considerable degradation, especially at longer immersion times. This indicates that lithium incorporation may accelerate ion exchange and surface dissolution processes.

Nevertheless, the degradation values remain within acceptable limits for tissue engineering applications, where a controlled balance between material resorption and new bone formation is essential. These findings confirm the suitability of lithium-modified bioglass-ceramics for bioresorbable scaffold systems with tunable degradation behaviour.

**Figure 10:**
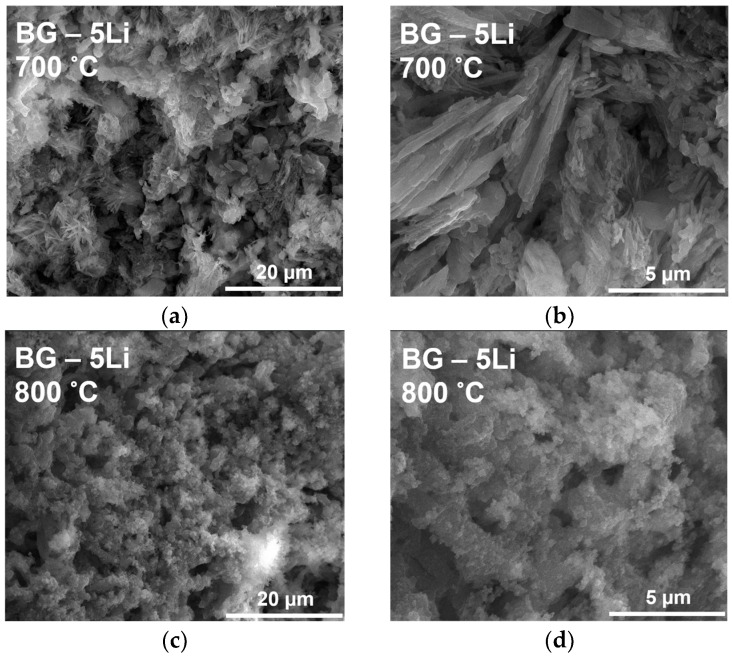
SEM micrographs of lithium-modified bioglass (5% Li) after 28 days of immersion in SBF. Images (**a**,**b**) correspond to the sample sintered at 700 °C, at 5000× and 20,000× magnification, respectively. Images (**c**,**d**) correspond to the sample sintered at 800 °C, also at 5000× and 20,000× magnification, respectively.

The SEM micrographs in Figure 10 illustrate the surface morphology of 5% lithium-modified bioglass after 28 days of immersion in SBF.

At 700 °C (Figure 10a,b), the surface reveals acicular and lamellar crystalline structures, indicative of active nucleation and growth of an apatite-like phase, commonly associated with in vitro bioactivity. In contrast, the 800 °C sample (Figure 10c,d) shows a denser, more compact matrix, likely due to lower surface reactivity or diminished ion mobility. Similar to previous research works on pure hydroxyapatite coatings, which typically exhibit continuous layers composed of plate-like crystals and irregular nanorods [61,62], our bioglass samples show comparable apatite-like features after SBF immersion, confirming that lithium supports the formation of a bioactive surface layer [63,64,65]. Overall, the microstructural differences confirm the influence of sintering temperature on surface bioactivity and mineral layer development.

**Figure 11:**
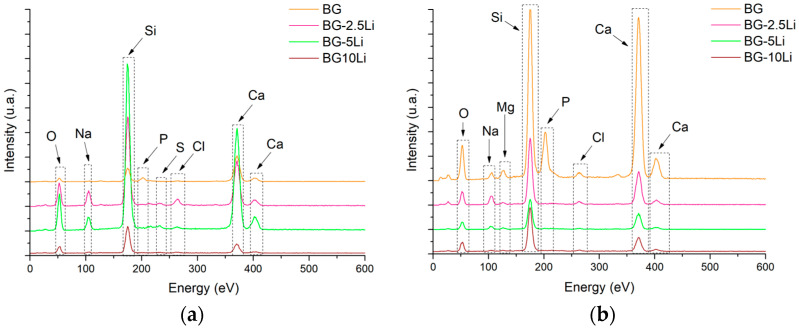
EDS spectra of bioglass (BG) and lithium-modified bioglass samples (2.5, 5 and 10 wt% Li) sintered at 700 °C (**a**) and 800 °C (**b**) after 28 days of immersion in SBF.

As shown in Figure 11, the EDS spectra reveal the elemental profile of the bioglass and lithium-modified samples after 28 days in SBF.

Key elements such as O, Na, Si, P, and Ca are consistently detected across all compositions, confirming the integrity of the silicate-phosphate glass network. The appearance of S and Cl peaks indicates the presence of sulphate and chloride species from the SBF, while the presence of Mg—especially in the 800 °C samples—suggests an increased ion exchange capacity at higher sintering temperatures. These findings point toward the formation of a bioactive surface layer, likely rich in calcium phosphates, and support the material’s potential for osseointegration.

### 3.4. Antibacterial Assessment

As highlighted by Zhang et al. [66], lithium-ion substitution enhances the antibacterial behaviour of bioactive glass systems, partly due to the strong affinity of Li^+^ ions for free water, which disrupts bacterial protein stability and leads to denaturation. This mechanism contributes to reduced bacterial viability and supports the use of lithium-substituted silicate networks in the development of bioactive materials with improved antimicrobial properties [67,68].

**Figure 12:**
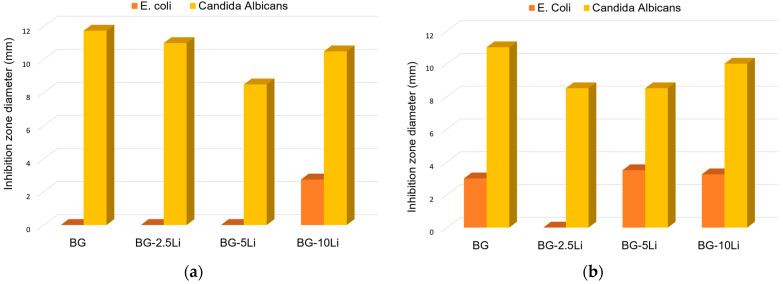
Antimicrobial activity of lithium-modified bioglass samples against *Escherichia coli* and *Candida albicans*, assessed by inhibition zone diameter (mm) after 24 h of exposure. Samples sintered at 700 °C (**a**) and 800 °C (**b**).

The antimicrobial activity of lithium-substituted bioglass, illustrated in Figure 12a,b, was evaluated using a diffusion-based inhibition zone assay.

**Figure 13:**
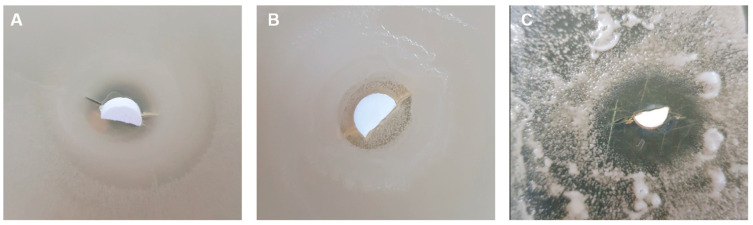
Photographic images of the inhibition zones formed around lithium-modified bioglass samples sintered at 800 °C, after 24 h of incubation. Images (**A**,**B**) show *Escherichia coli* inhibition zones for BG-10Li and BG, respectively, while image (**C**) shows the antifungal inhibition zone for BG-2.5Li against *Candida albicans*.

The clear halos (presented in Figure 13) around the samples indicate antimicrobial activity through ion diffusion into the surrounding agar medium.

All samples exhibited well-defined zones of inhibition against *Candida albicans*, with diameters exceeding 10 mm, indicating strong antifungal efficacy and high fungal susceptibility to the ionic dissolution products of the glass-ceramic surfaces. This may be related to the inhibitory effect of Li^+^ ions on *Candida* biofilm formation and budding, processes known to be disrupted by lithium at the cellular signalling level. In contrast, the antibacterial response against *Escherichia coli* was more limited, suggesting lower microbial sensitivity in this *Gram*-negative strain. However, previous studies indicate that lithium may also interfere with the regulation of ion transport genes in *E. coli* at the genomic level, providing an additional possible mechanism beyond ionic membrane destabilization. Notably, the 10Li samples showed improved bacteriostatic effect at both sintering temperatures, likely due to enhanced Li^+^ ion release, which may promote membrane destabilization or local pH modification. These results suggest that the antimicrobial effect is mainly due to the release of ions from the material into the surrounding medium, rather than direct contact with the microorganisms [67,68,69].

### 3.5. Cell Viability

**Figure 14:**
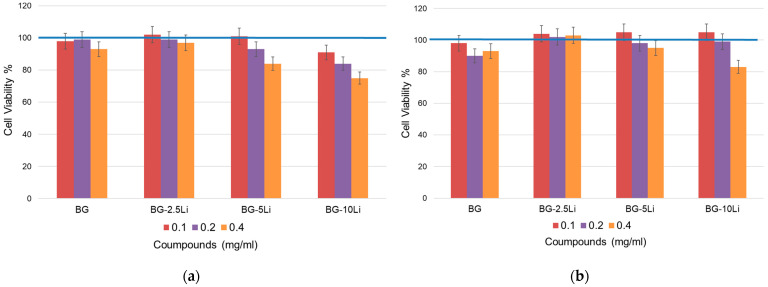
Cell viability (%) of MG-63 osteoblast-like cells after 48 h of exposure to lithium-modified bioglass at different concentrations (0.1, 0.2 and 0.4 mg/mL). Samples sintered at 700 °C (**a**) and 800 °C (**b**).

The cell viability results presented in Figure 14a,b demonstrate that all tested samples maintained values above 80%, indicating acceptable cytocompatibility with no signs of acute toxicity under the tested conditions.

At 700 °C (Figure 14a), a slight decrease in viability is observed with increasing lithium content and concentration, particularly at 0.4 mg/mL for BG-10Li. This suggests a dose-dependent response, possibly related to higher ion release from less stable matrices at lower sintering temperatures.

In contrast, the samples sintered at 800 °C (Figure 14b) exhibit slightly improved viability overall, with BG-2.5Li and BG-5Li maintaining values close to or above 95% across all concentrations. These results indicate that higher sintering temperatures may enhance biocompatibility by promoting matrix stability and reducing the release of potentially cytotoxic ions. Overall, the lithium-modified bioglass samples show promising behaviour for biomedical applications, especially when processed under optimized thermal conditions.

**Figure 15:**
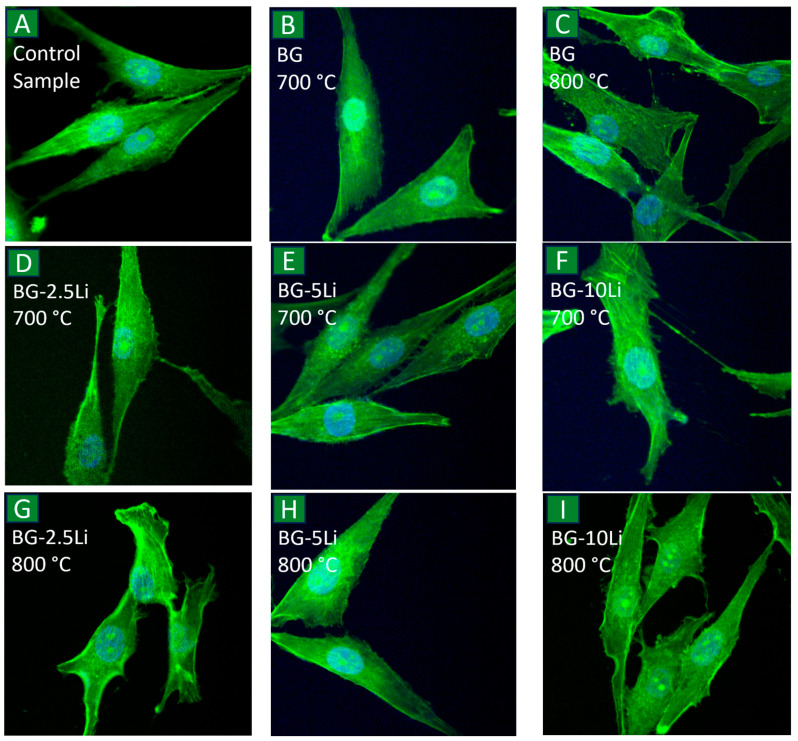
Fluorescence microscopy images of MG-63 osteoblast-like cells after 48h of incubation. Image (**A**) represents the control group (untreated cells), while (**B**,**C**) show cells exposed to bioglass (BG) sintered at 700 and 800 °C. Images (**D**–**F**) correspond to lithium-modified bioglass samples with 2.5, 5 and 10 wt% Li sintered at 700 °C, and images (**G**–**I**) show the same compositions sintered at 800 °C. All images were acquired using a 40× objective.

Figure 15 presents fluorescence microscopy images of MG-63 cells stained to visualize the nucleus and actin cytoskeleton, in order to assess the potential morphological impact of the tested materials.

Figure 15A–C show control cells and cells treated with BG at 700 and 800 °C, respectively. The cells maintain typical osteoblastic features, including elongated bodies, triangular morphologies, and filopodia-like extensions. Well-defined actin filaments aligned along the cell axis and terminating in focal adhesion points, as well as uniformly shaped oval nuclei, confirm that no morphological damage occurred [70,71]. These observations support the MTT data and indicate preserved cell functionality [72,73,74].

Similarly, cells exposed to lithium-substituted samples (Figure 15D–I) also retain normal morphology, even at the highest concentration tested (0.4 mg/mL). The cytoskeleton remains intact, and nuclei appear unaffected, suggesting that lithium incorporation does not induce cytotoxic effects. These findings confirm the biocompatibility of the developed materials and support their potential use in bone tissue engineering applications [70,74,75].

## 4. Conclusions

This study demonstrates that presence of lithium oxide in 47.5S5 bioglass composition (47.5 wt% SiO_2_, 23.75 wt% CaO, 23.75 wt% Na_2_O, and 5 wt% P_2_O_5_), combined with sintering at 700 and 800 °C, leads to the development of bioactive glass-ceramics with enhanced structural, mechanical, and biological performances. XRD, Raman, and SEM analyses confirmed the formation of crystalline phases such as NaLiSiO_4_ and Li_2_SiO_3_, particularly in the samples sintered at 800 °C, along with denser and more compact microstructures. The 5% Li composition exhibited the best balance between low porosity, high apparent density, and superior compressive strength (~42 MPa), making it a promising candidate for bone regeneration. Moreover, the formation of apatite-like surface layers after 28 days of SBF immersion—characterized by plate-like crystals and irregular nanorods—further confirms the bioactive nature of the materials.

From a biological perspective, lithium-modified bioglass-ceramics showed high cytocompatibility, with cell viability consistently above 80% under all conditions tested. Fluorescence microscopy confirmed that MG-63 cell morphology and cytoskeletal integrity remained unaffected. The antimicrobial evaluation revealed stronger inhibition against *Candida albicans*, while *Escherichia coli* exhibited lower sensitivity, although inhibition improved with higher lithium content. This may be attributed to mechanisms such as membrane destabilization, local pH changes, and genomic-level interference with ion transport regulation. Overall, lithium-modified glass-ceramics present a favourable combination of mechanical strength, controlled degradation, and moderate antimicrobial activity, making them strong candidates for future biomedical applications, particularly in bone tissue engineering.

## Figures and Tables

**Table 1:** Chemical composition of Li-modified bioactive glasses.

BG Type *	Composition (wt%)				
	SiO_2_	P_2_O_5_	Li_2_O	CaO	Na_2_O
BG	47.5	5	0	23.75	23.75
BG-2.5Li	47.5	5	2.5	21.25	23.75
BG-5Li	47.5	5	5	18.75	23.75
BG-10Li	47.5	5	10	13.75	23.75

* The sample codes indicate the degree of lithium oxide (Li_2_O) substitution of CaO. The reference material without lithium is labelled as “BG”, while the variants “BG-2.5Li”, “BG-5Li”, and “BG-10Li” denote the incorporation of 2.5, 5 and 10% Li_2_O.

## Data Availability

The original contributions presented in this study are included in the article. Further inquiries can be directed to the corresponding author.
